# Supplementary material for: Development and validation of an asthma self-knowledge questionnaire
Source: PLoS One. 2025 Oct 31;20(10):e0333760. doi: 10.1371/journal.pone.0333760 (PMC12578227; doi:10.1371/journal.pone.0333760)
Supplement: S1 Table — (PDF) [file pone.0333760.s001.pdf]

BRONCHIAL ASTHMA KNOWLEDGE QUESTIONNAIRE
(FINAL VERSION after exclusion of three initial questions)

Please read each statement with attention and state your opinion by placing an X in the square corresponding to the concordance level, in accordance with the legends below:

1	2	3	4	5
I fully disagree	I partially disagree	I neither agree nor disagree	I parcially agree	I fully agree

Example:

	1	2	3	4	5
**The word ASTHMA has six letters.**					×

	1	2	3	4	5
**1. Asthma is a chronic disease which persists even during periods without symptoms.**					
**2. Asthma begins more frequently in children or young adults than in the elderly.**					
**3. With good medical follow up, most asthmatics can lead a normal life.**					
**4. When asthma is not treated, it us a disease that may kill.**					
**5. In an asthmatic patient, an episode of flu may trigger an asthma bout.**					
**6. People with allergies are more likely to have asthma than people without allergies.**					
**7. The airways of the lungs (bronchi) are inflamed in asthma.**					
**8. The more inflamed bronchial airways are, the more severe is asthma likely to be.**					
**9. Some asthma symptoms are due to narrowing of the bronchi (lung airways).**					
**10. Coughing frequently may be a symptom of asthma.**					
**11. Asthma causes episodes of shortness of breath, but these are not really dangerous.**					
**12. An asthmatic patient should always go to an Emergency Department whenever he/she has mild shortness of breath.**					

	1	2	3	4	5
**13. An asthmatic patient who needs to use his/her rescue inhaler (p.r.n.) for asthma bouts many times weekly has his/her asthma controlled.**					
**14. Asthma can be well controlled in terms of symptoms.**					
**15. An asthma bout can be resolved by taking an anti-allergic pill.**					
**16. Hand tremor can be a side effect of rescue medication used to in asthma bouts.**					
**17. An asthmatic patient should hold his/her breath for 10 seconds after each inhaler medication inhalation.**					
**18. Asthmatic patients do not need to take medication outside of asthma bouts.**					
**19. Avoiding exposure to situations that may trigger an asthma bout, such as tobacco smoke and house dust, may improve asthma control.**					
**20. Asmatic patients should avoid doing any type of physical exercise.**					
**21. Certain sports, such as swimming, are better for asthmatic patientss.**					
